# Anti-Diabetic Effect of Soy–Whey Dual-Protein on Mice with Type 2 Diabetes Mellitus Through INS/IRS1/PI3K Signaling Pathway

**DOI:** 10.3390/foods14122115

**Published:** 2025-06-16

**Authors:** Na Li, Hu Li, Duo Feng, Mengjie Li, Di Han, Tianxin Liu, Jing Wang

**Affiliations:** Institute of Food and Nutrition Development, Ministry of Agriculture and Rural Affairs, Beijing 100081, China; 15910807606@163.com (N.L.); lihu01@caas.cn (H.L.); 15525926785@163.com (D.F.); limj0804@126.com (M.L.); handi87@126.com (D.H.); liutianxin2022@163.com (T.L.)

**Keywords:** dual-protein, type 2 diabetes, insulin resistance, mechanism of action, gut microbiota

## Abstract

The effects of soy protein and whey protein supplementation on glycemic control show inconsistency, and the mechanisms underlying the impact of a high-protein diet on blood glucose regulation remain unclear. This study aimed to explore the impact of a dual-protein (DP) blend comprising soy protein isolate (SPI) and whey protein concentrate (WPC), processed through high-pressure homogenization, on mice with Type 2 diabetes mellitus (T2DM) and its potential mechanisms. In the in vitro experiments, an insulin-resistant (IR) HepG2 cell model was treated with DP, resulting in a significant enhancement of glucose uptake and upregulation of IRS1 and GLUT4 expression. For the in vivo experiments, male C57BL/6J mice were randomly assigned into four groups (n = 6) based on body weight: normal control, T2DM model group, Metformin-treated group, and DP-treated group. Following a 5-week feeding period, Metformin and DP significantly reduced levels of blood sugar, AUC, TC, TG, and LDL-C in T2DM mice. Additionally, TP and ALB levels in the DP group were notably higher in the model group. In the liver and pancreas, DP alleviated histopathological changes and promoted liver glycogen synthesis in T2DM mice. Moreover, the levels of IRS1 and PI3K in the livers of mice in the DP group were significantly higher than those in the model group. Compared with the model groups, DP significantly reduced the expression of CD45 and increased the expression of CD206 in the pancreas of mice. Furthermore, 16S rRNA analysis revealed that DP altered the composition of the gut microbiota in diabetic mice, increasing the relative abundance of *Lactobacillus*, *Parvibacter*, and *Lactobacillaceae*. This suggested that DP could alleviate functional metabolic disorders in the gut and potentially reverse the risk of related complications. In conclusion, soy whey dual-protein may have an effective nutritional therapeutic effect on T2DM mice by regulating lipid metabolism, the INS/IRS1/PI3K signaling pathway, and gut microbiota.

## 1. Introduction

Type 2 diabetes mellitus (T2DM) is a metabolic disorder syndrome characterized by hyperglycemia resulting from defective insulin secretion, which can induce chronic damage and functional impairment in tissues such as blood vessels, kidneys, and the nervous system [1]. Currently, oral hypoglycemic medications, including metformin, sulfonylureas, and α-glucosidase inhibitors, remain effective methods for controlling the progression of T2DM [2,3]. However, long-term use of these drugs by T2DM patients can lead to numerous adverse effects, such as gastrointestinal intolerance or metabolic acidosis [4]. Notably, dietary and nutritional interventions have proven to be effective in managing the condition of T2DM patients and improving their quality of life, representing one of the emerging and promising therapeutic approaches with minimal side effects for the treatment of T2DM [5].

Protein supplements or high-protein diets have been proven to effectively improve blood sugar regulation [6]. Dietary proteins and amino acids influence insulin sensitivity and regulate glucose transport and utilization in the body through a series of complex biochemical reactions [7]. Studies have shown that whey protein could reduce risk factors for T2DM by enhancing satiety, promoting insulin secretion to lower postprandial blood glucose levels, and facilitating weight loss, thereby preventing obesity and the onset of T2DM [8]. However, the specific mechanisms remain unclear. Additionally, soy protein and peptides could act on various molecular targets, such as α-glucosidase, α-amylase, and dipeptidyl peptidase IV, as well as intracellular signaling pathways in multiple organs, to maintain glucose homeostasis and improve impaired glucose and lipid metabolism [9]. Although high-quality proteins like soy protein and whey protein have long been widely used in nutritional interventions, only a limited number of studies have reported their effects on metabolic diseases, with inconsistent conclusions and mechanisms that still require further exploration [10,11,12,13,14,15]. Dual-protein foods are a new dietary concept proposed in recent years with the aim of supplementing high-quality protein. It is of great significance to promote the combination of protein resources from different sources by using high-tech method means to improve their nutritional quality [16,17]. High-pressure homogenization (HPH) is widely used in the food industry to produce emulsified foods, such as soft drinks, milk, and condiments. During the high-pressure homogenization process, protein molecules are exposed to cavitation, shearing, turbulence, and heating in a short period of time, which can disrupt protein aggregates and change protein structure, thereby affecting their solubility and other functional properties [18]. Our previous research found that HPH could improve the functional properties of proteins by altering the structure of soy–whey dual-proteins [19]. However, whether the alteration of protein function will have an intervention effect on T2DM remains to be verified.

The gut microbiota plays a crucial role in the dynamic ecosystem of the human body, and its composition can be influenced by numerous factors, such as the host and the environment. Numerous studies have confirmed that imbalances in gut microbiota are not only closely associated with intestinal diseases but also significantly linked to metabolic disorders such as diabetes [20,21]. Therefore, modulating the gut microbial community is considered an important aspect of preventing or slowing down the progression of T2DM. Studies have shown that high-protein diets or protein-based nutritional interventions could influence the composition and abundance of gut microbiota. This is primarily attributed to the proteolytic fermentation of undigested proteins by bacterial proteases in the colon. Subsequently, metabolites released by the gut microbiota can affect host physiology [22]. Our previous research had demonstrated that DP supplementation increases the diversity of gut microbiota and alters microbial abundance patterns in patients undergoing allogeneic hematopoietic stem cell transplantation. It could also alleviate osteoporosis by modulating the gut microbiota, indicating its potential for regulating gut microbial communities [23,24]. Therefore, we hypothesized that DP nutritional intervention could reverse T2DM by modulating the gut microbiota.

Herein, this study investigated the effects of DP on alleviating insulin resistance (IR) and T2DM both in vitro and in vivo, and explored the role of gut microbiota in improving metabolic health under DP intervention. We induced insulin resistance in HepG2 cells (IR-HepG2) and tested the efficacy of DP in ameliorating cellular insulin resistance. Subsequently, a T2DM mouse model was established using a high-fat diet (HFD) combined with streptozotocin (STZ) to investigate the effects of DP on improving glucose, lipid, and protein metabolism. Additionally, 16S rDNA amplicon sequencing was employed to further explore the composition of gut microbiota and key regulatory factors underlying the improvement of metabolic health by DP.

## 2. Materials and Methods

### 2.1. Chemicals and Reagents

SUPRO 787 IP SPI (protein content, 90%) was obtained from IFF Inc. (Shanghai, China). WPC (protein content, 80%) was obtained from Arla Foods (DANEMARK).

Insulin was purchased from Shanghai Aladdin Biochemical Technology Co., Ltd. (Shanghai, China) and metformin from Haohong Biopharmaceutical Technology Co., Ltd. (Shanghai, China). 3-(4,5-dimethylthiazol-2-yl)-2,5-diphenyltetrazolium bromide (MTT) and sodium dodecyl sulfate (SDS) were purchased from Beyotime (Shanghai, China). Glucose kit (glucose oxidase method) was purchased from Nanjing Jiancheng Biotechnology Co., Ltd. (Nanjing, China).

STZ and metformin was purchased from Sigma-Aldrich (St. Louis, MO, USA). IRS1 Rabbit mAb, PI3K Rabbit mA, and GLUT4 Rabbit mA were purchased from Cell Signaling Technology(Boston, US). Goat Anti-Rabbit IgG H&L (Alexa Fluor^®^ 488) (ab150077) and Goat Anti-Rabbit IgG H&L (Alexa Fluor^®^ 555) (ab150078) were purchased from Abcam(Cambridge, Britain). 2-(4-amidinophenyl)-1H-indole-6-carboxamidine (DAPI) was purchased from Solarbio, Beijing, China. PE anti-mouse CD11b antibody, FITC anti-mouse CD45 antibody, PE anti-mouse CD3 antibody, FITC anti-mouse CD8 antibody, and APC anti-mouse CD4 antibody were purchased from Biolegend, USA. All other reagents were analytical reagents from chemical companies in China.

### 2.2. Soy–Whey Dual-Protein Solution Sample Preparation

Our team previously conducted a study on the application of high-pressure homogenization to dual-protein [19]. On this basis, the DP optimization treatment is carried out. Soy protein isolate (SPI) and whey protein concentrate (WPC) were weighed, respectively, and according to the team’s preliminary results, they passed through the planetary ball mill at a ratio of 1:1. The rotational speed was set at 500 rpm/min, the intermittent time was 30 min, and the mixed protein powder was obtained by grinding and mixing for 8 h. Then, the DP powder was weighed and then dissolved in deionized water to prepare a 6% (*w*/*w*) DP solution. The solution was stirred magnetically for 1.5 h. Then, the container was sealed with plastic wrap and allowed to hydrate in a refrigerator at 4 °C for 10 h. After standing and returning to room temperature, the filtrate was homogenized under 60 MPa high pressure through 60 mesh sieves, and each pressure cycle was three times. After homogenization, the pH of the solution was adjusted to 7 to obtain the final DP sample. The amino acid profile of DP is detailed in Supplementary Table S1.

### 2.3. Cell Experiments

#### 2.3.1. Cell Culture

Human hepatocellular carcinoma cell line HepG2 cells were acquired from Shanghai Cell Bank of the Chinese Academy of Sciences. The cell lines were cultured in DMEM, which contained 10% fetal bovine serum (FBS; Hyclone, Thermo Scientific, Waltham, MA, USA), penicillin (100 U/mL), and streptomycin (100 μg/mL) (Gran Island, NY, USA). The incubator conditions were set at 37 °C and 5% CO_2_. A 0.25% trypsin was used for digestion and passage.

#### 2.3.2. Cytotoxicity

A total of 5 × 10^3^ cells/well were seeded on 96 well plates and allowed to adhere overnight. The cells were treated with increasing concentrations of the sample diluted in cell media achieving a total volume of 100 μL. The cells were incubated with Met and increasing concentrations of DP at 37 °C for 24 h. Next, the medium with serum was replaced with fresh medium containing (3-(4,5-dimethylthiazol-2-yl)-2,5-diphenyltetrazolium bromide) (MTT, 10 µL of a 5 mg/mL solution in PBS buffer) and the cells were further incubated for 4 h. 10% SDS solution was then added (100 µL/well) and the plates were kept in the dark for an additional 12 h. Absorption measurements were performed on a Bio-Rad plate reader at 570 nm (peak absorbance) and at 650 nm (background absorbance).

#### 2.3.3. Glucose Uptake

HepG2 cells were seeded in 96-well plates in DMEM supplemented with 10% (*v*/*v*) FBS. The density of the cells in each plate was 5 × 10^3^ cells/well. Preliminary experiments were conducted to establish insulin resistance model in HepG2 cells in accordance with Zheng et al. [25]. We removed the original medium when the cells became adherent and replaced it with DMEM containing 10^−6^ mol/L insulin. The model of insulin resistance (IR-HepG2) was established after 24 h. Control group, insulin resistance model group (IR), metformin (Met) group (0.03 mg/mL), and DP group (1.00, 5.00, 10.00 mg/mL) were set up in the experiment, with three parallel cells in each group. The culture was continued for 24 h, and then the glucose content in the culture medium supernatant was determined by glucose detection kit.

#### 2.3.4. In Vitro Fluorescence Staining

HepG2 cells were seeded on 24-well plates (1 × 10^4^ cells/well) and allowed to adhere overnight. The IR-HepG2 model was established after 24h induction with 10^−6^ mol/L insulin. The cells were then treated with DP (10 mg/mL) and metformin (0.03 mg/mL) for 24 h. PBS served as control treatment. The above cells were washed with PBS and further incubated with IRS1 Rabbit Monoclonal Antibody for 2 h at 37 °C. Subsequently, the cells were incubated with Goat Anti-Rabbit IgG H&L (Alexa Fluor^®^ 488) for 1 h at 37 °C. The expression of IRS1 inside the cells was observed by CLSM.

#### 2.3.5. Western Blot

HepG2 cells were seeded in 6-well plates (1 × 10^6^ cells/well) and allowed to adhere overnight. The IR-HepG2 cell model was established after 24 h induction with 10^−6^ mol/L insulin. The cells were treated with DP (10 mg/mL) and metformin (0.03 mg/mL) for 24 h. After washing the cells with cold PBS three times, RIPA lysis buffer containing protease and phosphatase inhibitors was added to each well. The proteins of cells were extracted through centrifuge at a speed of 12,000 rpm for 5 min. Protein content quantification was carried out by the BCA protein assay kit. Then, the electrophoreses process was conducted through SDS-PAGE by a gel-electrophoretic apparatus (Bio-Rad mini, Hercules, CA, USA), and the proteins were transferred to the PVDF films and incubated with the antibodies against various proteins overnight on a shaker at 4 °C. Subsequently, the PVDF films were washed five times and incubated with HRP conjugated antibodies for 1 h. The western blot images were obtained by Amersham Imager 600 (AI600, General Electric Co., Ltd., Boston, MA, USA) with 300 μL of ECL chemiluminescent reagent (Beyotime Biotechnology Co., Ltd., Shanghai, China) added on the top of the membrane.

### 2.4. Animal Experiments

Six-week-old male C57BL/6J mice (approximately 16 to 18 g) were purchased from Sibeifu (Beijing) Biotechnology Co., Ltd. (Beijing, China). Mice were housed no more than six per cage under standard laboratory conditions (12 h light/dark cycle at room temperature [approximately 22–24 °C]) with free access to clean water and a standard diet. The entirety of animal experiments conducted received approval from the Animal Management and Ethics Committee of the Institutional Animal Care and Use Committee, Institute of Food and Nutrition Development, Ministry of Agriculture and Rural Affairs, as per the stipulated license number 20240315-05.

**Figure 1:**
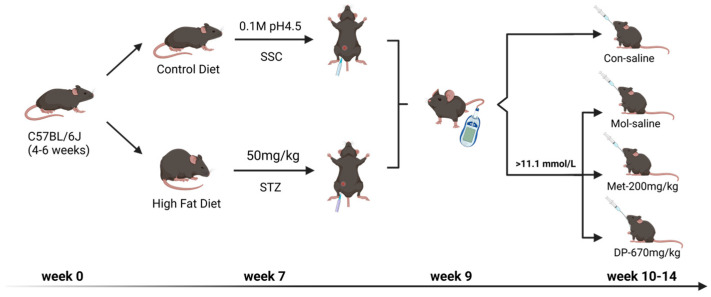
Animal experimental design. Con: normal control mice; Mol: diabetic model mice; Met: diabetic mice treated with metformin (200 mg/kg); DP: diabetic mice treated with DP (670 mg/kg). Figure created with Biorender.com.

The whole animal experimental design is summarized in Figure 1. All C57BL/6J mice were adaptively administered with common chow for a week and further replaced with a high-fat diet (HFD) for 6 weeks. The ingredients of HFD were 66.5% common diet, 20% sucrose, 10% fat from lard, 2.5% cholesterol, and 1% sodium deoxycholate (SPF (Beijing, China) BIOTECHNOLOGY Co., Ltd. (Fuxin, China)).

Streptozotocin (STZ) was intraperitoneally injected into mice on days 1 and 3 of the 7th week at a dose of 50 mg/kg. Mice were required to fast for 12 h before STZ injection. STZ was dissolved in Citrate Buffered Saline (SSC) (pH 4.2–4.5). Notably, it required temporary preparation for use and protection from light and was placed in an ice bath during the injection. Fasting blood glucose (FBG) level was measured in the 9th week, and mice with FBG > 11.1 mmol/L were considered to be successfully modeled with diabetes. Furthermore, they were randomly divided into a T2DM model (Mol) group, metformin (Met) group, and dual-protein (DP) group based on the FBG level, and C57BL/6J mice reared on common chow for the same period comprised the normal control (Con) group (6 mice/group). Mice in the Met group were given metformin orally (200 mg/kg/day), and mice in DP group were given DP solution orally (670 mg/kg/day). Equal amounts of saline were administered by oral gavage to both the Con and Mol groups mice for 5 weeks consecutively.

#### 2.4.1. Body Weight, FBG, and Glucose Tolerance Test (OGTT)

Body weight and FBG were measured every week during the experimental period. In the last week of the experimental period, the animals were subjected to an oral glucose tolerance test. In brief, mice were fasted for 12 h and administered an oral glucose load (2 g/kg of body weight), and then the blood glucose level was measured using a glucometer (Sinocare) at 0, 30, 60, 90, and 120 min, respectively. The areas under the curve (AUCs) of blood glucose levels were calculated to evaluate glucose tolerance.

#### 2.4.2. Analysis of Serum Biochemistry

Total cholesterol (TC), triacylglycerols (TG), high-density lipoprotein cholesterol (HDL-C), low-density lipoprotein cholesterol (LDL-C), total protein (TP), and albumin (ALB) were examined by an Automatic biochemical analyzer (Chemray 800, Shenzhen, China).

#### 2.4.3. Detection of Immunohistochemistry and Immunofluorescence

Livers and pancreas were collected and a slice of each one was fixated with 4% paraformaldehyde. Liver and pancreas were stained with hematoxylin and eosin (H&E) and the liver was stained with Periodic-acid Schiff (PAS) staining. The histological images were taken using a CLSM (LSM-800, ZEISS, Oberkochen, Germany).

For immunofluorescence analysis, frozen sections of pancreas were incubated for 14 h at 4 °C with a primary antibody for insulin (ab7842, Abcam), glucagon (ab10988, Abcam), and then with secondary antibodies at room temperature for 2 h. Slides were observed under a laser scanning confocal microscope. The area of cells positive for insulin antibody in the islets were counted and used to calculate the positive cell ratio.

Immunohistochemistry (IHC) was used to analyze the expression of IRS1 and PI3K in the mouse livers. Specifically, the wax-embedded livers were cut into 5 μm sections. We used anti-mouse IRS1 (Servicebio, Wuhan, China) and PI3K (Servicebio, Wuhan, China) antibody (1:100) at 4 °C overnight for IHC staining. All slices were counterstained with hematoxylin at 25 °C for 1 min. microscopic images were obtained with a light microscope. Three random areas from each sample were chosen and analyzed using Image J (National Institutes of Health, Bethesda, MD, USA).

#### 2.4.4. Detection of CD45 in Pancreas Slices by CLSM

Following the previously described treatment, pancreas slices from each group of animals were collected. The tissue was incubated with CD45+-specific fluorescent probe. The presence of CD45+ T cells were assessed using CLSM (LSM-800, ZEISS, Germany).

#### 2.4.5. Detection of CD45 T Cells in Pancreas by Flow Cytometry

Fresh pancreatic tissues were collected for antidiabetic immune response analysis via FACS. Briefly, samples were dissociated into single-cell suspensions, and then red blood cells were removed with red blood cell lysing buffer (Beyotime). After that, samples were blocked with 0.1% BSA in PBS followed by incubation with relevant antibodies for 1 h at room temperature. For characterizing T cells and leukocyte in pancreas, cells were stained by anti-CD3-PE, anti-CD4-PC5.5, anti-CD8-FITC, and anti-CD45-FITC. All the antibodies used above were all purchased from Bio Legend. Flow cytometric data acquisition was performed with CytExpert-2.6 software, and the data were processed using FlowJo-v10 software. Data were expressed as mean ± SD (n = 3).

#### 2.4.6. 16S rDNA Sequencing Analysis

In this experiment, Illumina novaseq6000 sequencing platform was used to amplify and sequence the V3-V4 variable region. First, the mouse feces DNA was extracted by the kit, and the purity and concentration were detected. After the V3–V4 variable region was selected, PCR amplification was performed using a specific primer with Barcode and a high-fidelity DNA polymerase. PCR products were detected by 2% agarose-gel electrophoresis, and target fragments were recovered by using the Quant-iT PicoGreen dsDNA Assay Kit. Then, the recovered products were detected and quantified by FLx800 fluorescence quantification system. The TruSeq Nano DNA LT Library Prep Kit was used to construct the library. In order to ensure that the library quality met the standards of on-machine sequencing, Agilent Bioanalyzer 2100 and Promega QuantiFluor were used for quality inspection, and after passing the inspection, on-machine sequencing was performed.

Raw data obtained by sequencing has a certain proportion of dirty data. In order to make the results of information analysis more accurate and reliable, quality control analysis such as quality filtering, noise reduction, splicing, and chimera removal were performed on the raw data by using Qiime2 default parameters, and sequences with abundance less than 10 are filtered out (all samples are summed up), so as to obtain ASVs. Based on the flattened ASVs, ASV diversity index analysis and sequencing depth detection could be performed. Based on taxonomic information, statistical analysis of community structure could be performed at various taxonomic levels.

Sequence analysis was carried out using UPARSE software package and UPARSE-OTU and UPARSE-OTUref algorithms. Sequences with similarity ≥97% were assigned to the same OTU. We selected representative sequences for each OTU and label each representative sequence with classification information using the RDP classifier.

### 2.5. Statistical Analysis

Data were expressed as mean ± SD. All the data were analyzed using GraphPad Prism 9.0 (GraphPad Software, Inc. La Jolla, CA, USA). Statistical significance was analyzed using a one-way analysis of variance (ANOVA) followed by the Bonferroni test. Data were considered statistically significant for *p* < 0.05.

## 3. Result

### 3.1. Effects of DP on the Insulin Resistance (IR)-HepG2 Cell Model

**Figure 2:**
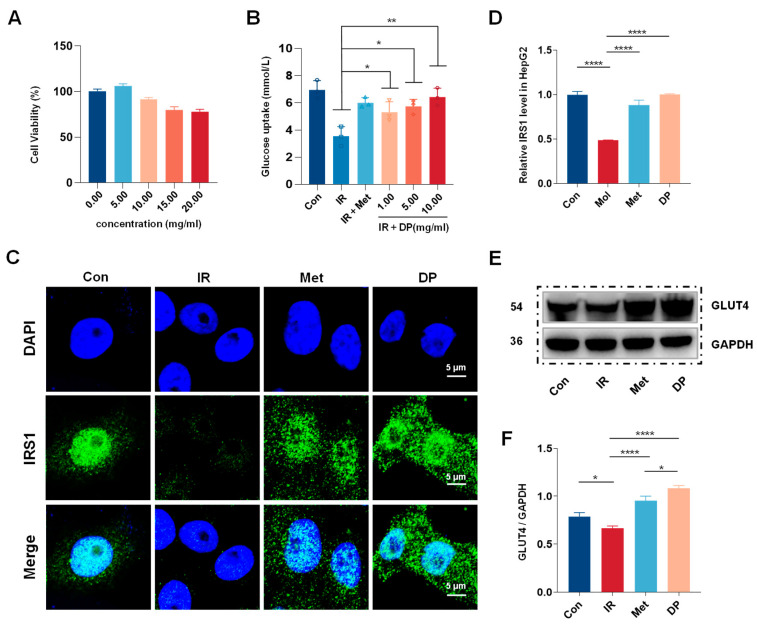
In vitro DP promotes glucose uptake and activates IRS1/GLUT4 pathway in HepG2 cells. (**A**) Cell viability of HepG2 treated with DP in 24 h. (**B**) Glucose intake in different treatment groups in 24 h. (**C**) Representative CSLM images of IRS1 expression on HepG2 treated with metformin (0.03 mg/mL) and DP (10 mg/mL). The cell nuclei were stained with DAPI (blue) and IRS1 were stained with Alexa Fluor^®^ 488 (green). (**D**) Relative IRS1 level in HepG2. (**E**) Western blot analysis to detect the expression levels of GLUT4 treated by metformin (0.03 mg/mL) and DP (10 mg/mL) for 24 h, and GAPDH was used as an internal reference protein. (**F**) Quantitative western blot analysis of GLUT4 by Image J software (n = 3, data are presented as mean ± SD). Statistical significances between every two groups were calculated via one-way ANOVA. * *p* < 0.05, ** *p* < 0.01, **** *p* < 0.0001.

HepG2 cells are commonly utilized for investigating hepatic insulin signaling pathways and glucose homeostasis in vitro. The cytotoxicity of DP on HepG2 cells was evaluated using the MTT assay after 24 h to establish its effective concentration (Figure 2A). Experimental groups were exposed to varying concentrations of DP (5, 10, 15, and 20 mg/mL), while a Con group remained DP-free. The findings demonstrated a biphasic effect of DP on cell growth and differentiation, with promotion at lower concentrations and inhibition at higher concentrations. Specifically, DP at 5 mg/mL enhanced HepG2 cell growth and differentiation, yielding a cell viability rate of 105.98% compared to the Con group. Conversely, at 10 mg/mL, DP initiated inhibition of HepG2 cell growth, resulting in a cell viability rate of 91.45%. These outcomes suggest that DP concentrations below 10 mg/mL have negligible impact on HepG2 cell viability.

To examine the impact of DP on glucose uptake and cell viability in insulin-induced HepG2 cells (IR-HepG2 model), we quantified glucose levels in the culture supernatant using a glucose oxidase assay kit and evaluated cell viability (Figure 2B). Our findings revealed that glucose uptake was 6.95 mmol/L in the Con group, whereas in the IR group treated with 10^−6^ M insulin, glucose uptake significantly decreased to 3.52 mmol/L (*p* < 0.01), confirming the successful establishment of the IR-HepG2 cell model. DP at concentrations of 1, 5, and 10 mg/mL significantly enhanced glucose uptake in IR-HepG2 cells compared to the IR group, with higher concentrations yielding greater improvements. Notably, at a DP concentration of 10 mg/mL, glucose uptake reached 6.43 mmol/L, surpassing the effect of the positive control metformin (0.03 mg/mL), albeit without statistical significance. These results indicate the development of insulin resistance in the IR group, while highlighting the potential of DP at specific concentrations to ameliorate glucose utilization in insulin-resistant HepG2 cells in a dose-dependent manner.

To further explore the mechanism of DP on HepG2 cells, we utilized laser scanning confocal microscopy (CLSM) to assess the expression of IRS1 in the IR-HepG2 cell model and conducted semi-quantitative analysis (Figure 2C,D). Our CLSM findings demonstrated a reduction in IRS1 protein levels in HepG2 cells to 57.30% of the Con group under insulin stimulation. After intervention with metformin (0.03 mg/mL) and DP (10 mg/mL), the expression levels of IRS1 protein were restored by 44.74% and 51.4%, respectively. These results indicate that DP enhances glucose uptake by elevating IRS1 expression, thereby significantly enhancing glucose metabolism. Furthermore, to evaluate the translocation of GLUT4 protein in the IR-HepG2 cell model, we assessed GLUT4 protein expression through western blotting (Figure 2E). All western blot data were normalized against GAPDH as a housekeeping protein control. Band intensities were quantified using ImageJ(x64) software (version 1.8.0_60) and presented as ratios of target protein to GAPDH (Figure 2F). Our data indicated a significant reduction in GLUT4 expression in the Mol group post insulin induction compared to the Con group. However, following a 24-h treatment with DP (10 mg/mL), GLUT4/GAPDH increased by 1.62-fold relative to Mol group (*p* < 0.01). These findings suggest that DP facilitates GLUT4 translocation in the IR-HepG2 cell model, leading to enhanced glucose transport into the cells and mitigation of insulin resistance.

### 3.2. Effects of DP on Blood Glucose, Body Weight, and Food Intake in T2DM Mice

**Figure 3:**
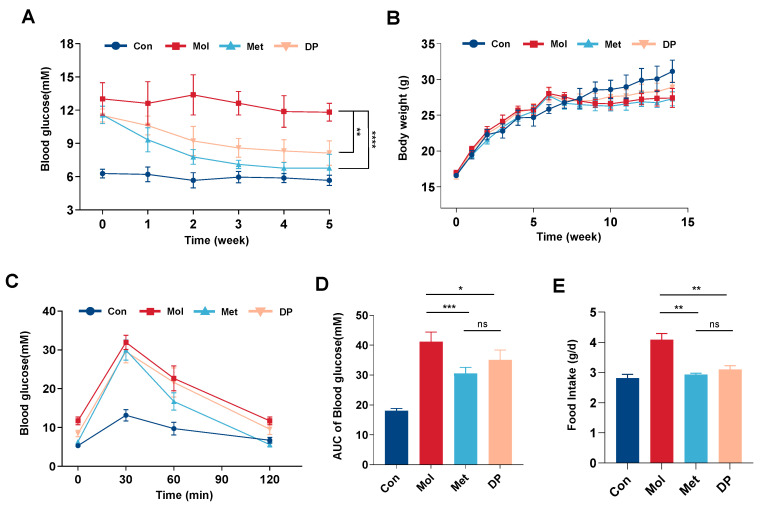
Effect of DP on glucose metabolism in T2DM mice. The weekly changes of blood glucose (**A**) and body weight (**B**) in T2DM mice during DP intervention. (**C**) OGTT test performed at the end of the experiment. All mice were fasted overnight, and then orally administered a 2.0 g/kg dose of glucose. The blood glucose levels were measured at 0, 30, 60, 90, and 120 min. (**D**) The area under the curve (AUC) of each group. (**E**) Food intake of mice after intervention (n = 6, data are presented as mean ± SD). Statistical significances between every two groups were calculated via one-way ANOVA. * *p* < 0.05, ** *p* < 0.01, *** *p* < 0.001, **** *p* < 0.0001.

To investigate the impact of DP on blood glucose levels in T2DM mice, fasting blood glucose (FBG) levels were measured weekly for five consecutive weeks (Figure 3A). Compared to the normal mice in the Con group, the FBG levels in T2DM mice were significantly elevated (*p* < 0.01) and exceeded 11.1 mmol/L, indicating the successful establishment of a stable T2DM mouse model induced by HFD/STZ. After five weeks of intervention with DP and metformin, the FBG levels in the Met group decreased more significantly (*p* < 0.01), dropping from 11.57 mmol/L to 6.78 mmol/L. Similarly, the FBG levels in the DP intervention group also showed a significant reduction (*p* < 0.01), decreasing from 11.52 mmol/L to 8.13 mmol/L.

Changes in body weight are shown in Figure 3B. Following six weeks of HFD feeding, the average body weight of the Mol group increased from 16.93 g to 27.98 g. Subsequent to the induction of the T2DM model, the mice displayed symptoms including polydipsia, polyphagia, polyuria, and weight loss. Their FBG levels surpassed 11.1 mmol/L, with random blood glucose levels exceeding 16.7 mmol/L, thereby confirming the successful establishment of the T2DM model. Weekly monitoring of body weight indicated that, in comparison to the Con group, the Mol group mice exhibited a consistent decline in weight. Following treatment with metformin and DP, their body weight commenced recovery and displayed a continuous increase. Of note, the body weight of the DP group mice was higher than that of the model group, suggesting that DP could ameliorate weight loss in mice with T2DM. Conversely, the weight gain rate in the Met group was lower than that in the other groups, possibly attributable to the gastrointestinal side effects associated with metformin.

The oral glucose tolerance test (OGTT) was utilized to assess fluctuations in blood glucose levels in mice over a 2 h period following the oral administration of a high dose of glucose (Figure 3C,D). The OGTT, along with the calculation of the area under the curve (AUC), serves as indicators of the glucose tolerance of the mice. To explore the impact of DP on abnormal glucose tolerance in HFD/STZ-induced T2DM mice, an OGTT was conducted on four mouse groups following five weeks of continuous intervention. Results from the OGTT indicated a noteworthy distinction in the rate of glucose level elevation within 30 min post-administration of a 20% glucose solution between the Con group and Mol group. The AUC was computed, revealing a significant reduction in the AUC with DP intervention compared to the Mol group (*p* < 0.05). Moreover, the Met group exhibited an even more substantial decrease in AUC (*p* < 0.01). These findings suggest that DP has the potential to ameliorate abnormal OGTT and AUC in T2DM mice, thereby enhancing their glucose tolerance. Furthermore, as depicted in Figure 3E, there was a notable decrease in food intake among T2DM mice (*p* < 0.01), indicating that both metformin and DP could effectively mitigate the heightened food consumption observed in T2DM mice. 

### 3.3. Effects of DP on Lipid and Protein Metabolism in Mice with T2DM

**Figure 4:**
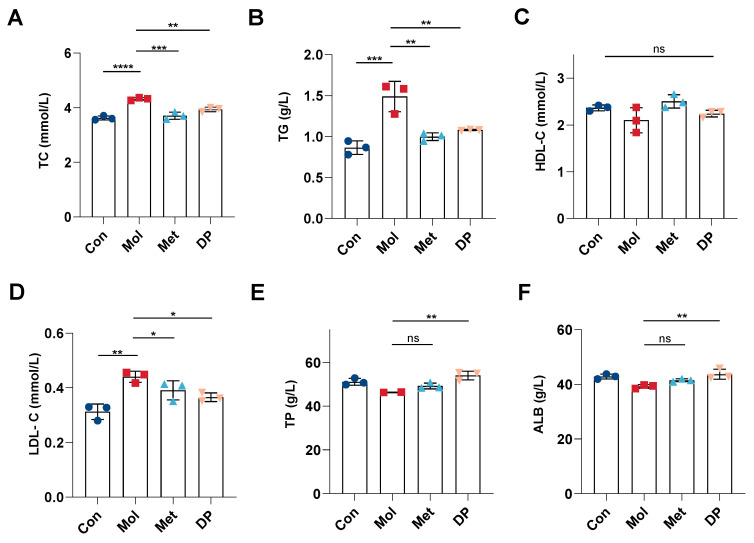
Effect of DP on lipid metabolism and protein metabolism in mice with T2DM. TC (**A**), TG (**B**), HDL-C (**C**), LDL-C (**D**), TP (**E**), and ALB (**F**) concentration in serum after intervention (n = 3, data are presented as mean ± SD). Statistical significances between every two groups were calculated via one-way ANOVA. * *p* < 0.05, ** *p* < 0.01, *** *p* < 0.001, **** *p* < 0.0001, ns—no significant difference.

Diabetes commonly co-occurs with disruptions in lipid and protein metabolism, with lipid metabolism intricately linked to insulin and glucose regulation in the body. Figure 4A–D illustrate the concentrations of TC, TG, HDL-C, and LDL-C. Our findings indicate that TC, TG, and LDL-C levels were markedly elevated in mice with T2DM compared to those in normal mice. Conversely, treatment with DP significantly decreased TC (*p* < 0.01), TG (*p* < 0.01), and LDL-C levels (*p* < 0.05), while no significant difference was observed in HDL-C levels.

In Figure 4E,F, protein metabolism indicators were assessed. In T2DM mice, compared to the Con group, serum levels of TP and ALB significantly decreased to 46.34 g/L and 39.22 g/L, respectively (*p* < 0.05). Following metformin intervention, TP concentration increased to 49.26 g/L, showing no significant difference compared to the Con and Mol groups. In the DP group, TP concentration rose to 54.03 g/L, significantly higher than in the Mol group (*p* < 0.01) and slightly higher than the control group’s 51.18 g/L. ALB levels in the metformin group increased to 41.46 g/L, with no significant difference compared to the model group. Conversely, ALB concentration in the DP group increased to 43.71 g/L, showing a significant difference compared to the model group (*p* < 0.01).

### 3.4. Effects of DP on Liver Injury in Mice with T2DM

**Figure 5:**
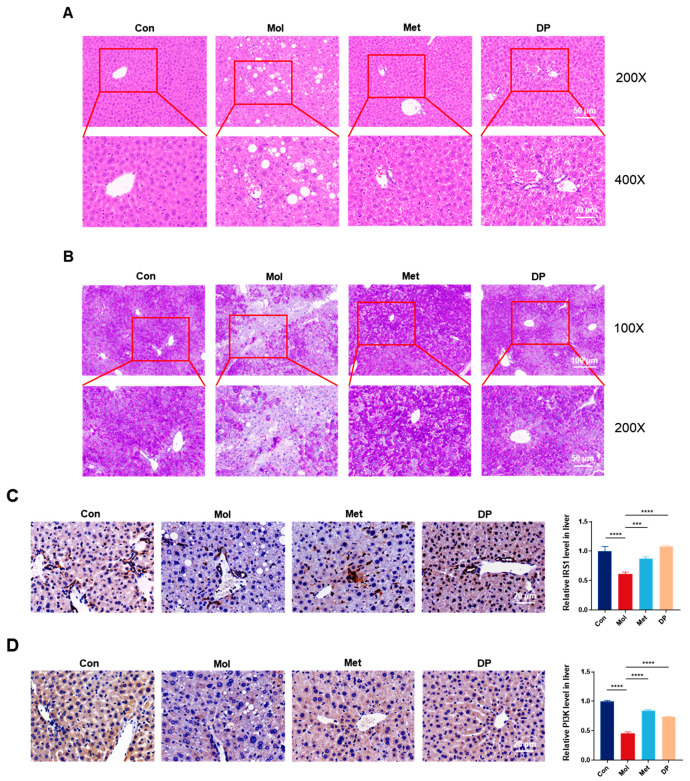
Effect of DP on liver histopathology and the relative expression of IRS1 and PI3K on liver. (**A**) HE staining study of liver tissues in each treatment group. (**B**) PAS staining of liver tissue of mice in each treatment group. Scale bar = 100 μm. (**C**) The relative expression of IRS1 on liver. Quantitative analysis was conducted using Image J software. (**D**) The relative expression of PI3K on liver. Scale bar = 20 μm. Quantitative analysis was conducted using Image J software (n = 3, data are presented as mean ± SD). Statistical significances between every two groups were calculated via one-way ANOVA. *** *p* < 0.001, **** *p* < 0.0001.

Hematoxylin and eosin (H&E) staining of liver tissue sections was conducted to assess the hepatoprotective effects of DP (Figure 5A). In healthy mice, liver histology displayed a standard lobular structure with hepatocytes encircling the central vein. Conversely, T2DM mice exhibited pronounced fat accumulation and extensive lipid vacuolation in the liver. The results suggest that DP supplementation markedly ameliorated histopathological alterations and microvesicular steatosis in the liver.

Periodic acid–Schiff (PAS) staining was utilized to assess the impact of DP on hepatic glycogen levels in the liver (Figure 5B). Following PAS staining, hepatic glycogen was observed in various shades of purple, while cell nuclei were stained blue. Liver tissue samples from the control group exhibited a consistent deep purple hue, indicating high glycogen levels in healthy mice. In contrast, liver tissue staining in the Mol group displayed a notably lighter shade. Following DP intervention, the intensity of glycogen staining in liver tissues increased, with the most pronounced staining observed in the metformin-treated group. These findings suggest that both metformin and DP supplementation can enhance hepatic glycogen levels, potentially by facilitating glycogen synthesis and modulating hepatic glycogen metabolism in T2DM mice, thereby contributing to improved glycemic control.

To further investigate the mechanism of DP intervention, we examined its impact on the expression of IRS1 (Figure 5C) and PI3K (Figure 5D) proteins through immunohistochemical staining, followed by semi-quantitative analysis using ImageJ software. In comparison to the Con group, the levels of IRS1 and PI3K proteins in the livers of Mol group mice were significantly decreased (*p* < 0.05), indicating impaired insulin receptor pathways and insulin signaling transduction in mice with T2DM. Following DP intervention, the expression levels of IRS1 and PI3K proteins were restored by 1.77-fold and 1.6-fold respectively, compared to the Mol group. These findings suggest that DP may mitigate IR by activating the IRS1/PI3K signaling pathway.

### 3.5. Effects of DP on Pancreatic Tissue in Mice with T2DM

**Figure 6:**
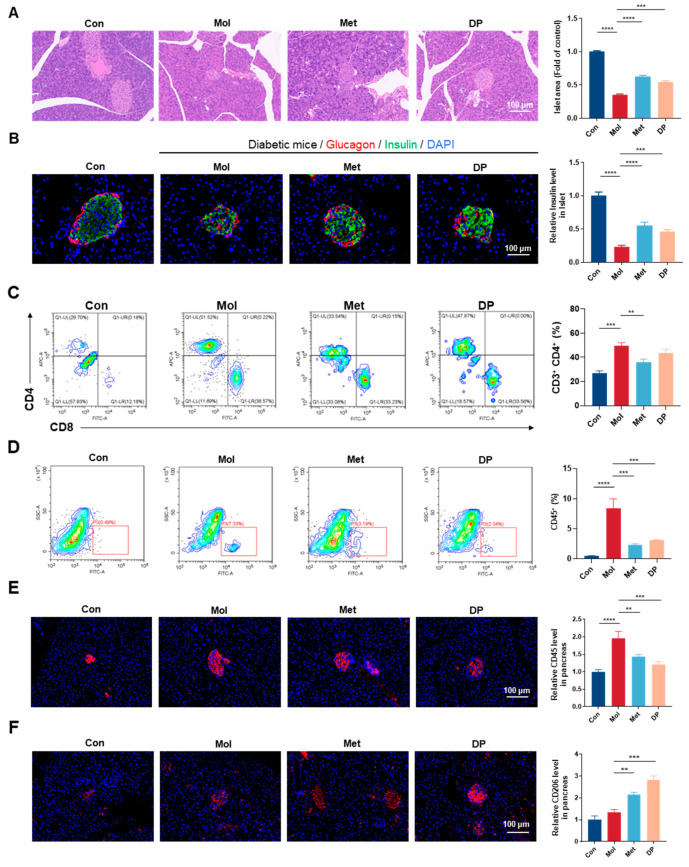
Effect of DP on pancreatic histopathology and in vivo anti-T2DM immune response of DP. (**A**) Representative images of HE-stained pancreas section. (**B**) Pancreatic insulin (Green) and glucagon (Red) CLSM. (**C**) Flow cytometric analysis of CD4+ and CD8+ cells gating on CD3+ cells in the pancreas. (**D**) Flow cytometric analysis of CD45 cells in the pancreas. (**E**) Representative CSLM images of CD45 cells (Red) in the pancreas. (**F**) Representative CSLM images of CD206 cells (Red) in the pancreas. The percentages of populations of cells in each group are presented as histograms. Scale bar = 100 μm (n = 3, data are presented as mean ± SD). Statistical significances between every two groups were calculated via one-way ANOVA. ** *p* < 0.01, *** *p* < 0.001, **** *p* < 0.0001.

Islet cell damage is a key feature of T2DM, and morphological changes in islets can be observed through H&E staining of pancreatic tissue. Figure 6A showed that in normal mice, islet cells exhibited a regular shape, clear boundaries, and an intact structure. Conversely, islets from T2DM mice exhibit notable changes, such as islet cell atrophy and morphological variations. A semi-quantitative analysis of islet cell area using ImageJ software indicates that both metformin and DP interventions effectively mitigate islet damage in T2DM mice to a comparable degree. There is a significant increase in the islet area compared to the Mol group (*p* < 0.01), with a trend towards improved islet structure integrity. These outcomes suggest that DP has the potential to ameliorate islet damage in T2DM.

In addition to causing islet cell damage, T2DM disrupts the regulation of insulin secretion, leading to insufficient insulin production [26]. Islet cells were stained using fluorescence-labeled insulin and glucagon antibodies, with nuclei counterstained using DAPI. As shown in Figure 6B, insulin (green fluorescence) was primarily localized in the central region of the islets, secreted by β-cells. Conversely, glucagon (red fluorescence) was mainly located in the peripheral region, secreted by α-cells. The results demonstrated that both metformin and DP interventions significantly promoted insulin secretion.

### 3.6. Effects of DP on Islet Inflammation Regulation in Mice with T2DM

To investigate the impact of DP intervention on immune inflammation in the islets of T2DM mice, we analyzed the expression of activated T-cell markers (CD4+ and CD8+) in pancreatic tissue of HFD/STZ-induced T2DM mice using flow cytometry (Figure 6C). Compared to the Con group, the Mol group exhibited a higher proportion of CD4+ cells (*p* < 0.01), accounting for 49.7%, confirming that T2DM promotes inflammatory progression. Following metformin and DP interventions, the proportion of CD4+ cells in the Met group significantly decreased to 36.13% (*p* < 0.01). The DP group also showed a reduction in CD4+ cells to 43.32%, although this was not statistically significant. These results suggested that DP may enhance immune regulation to some extent.

Figure 6D shows the flow cytometry results for CD45 in mouse pancreatic tissue. Compared to the Con group, the proportion of CD45+ cells in T2DM mice was significantly increased (*p* < 0.01). After DP intervention, the CD45+ ratio decreased from 8.44% to 3.12% (*p* < 0.01), while metformin intervention reduced the CD45+ ratio to 2.36% (*p* < 0.01). Similarly, immunofluorescence analysis of mouse pancreatic tissue showed a consistent trend (Figure 6E). These results indicated that DP could significantly suppress pancreatic tissue inflammation and reduce CD45+ cell infiltration.

To explore the effects of DP on macrophages, we performed immunofluorescence staining using CD206 to label M2 macrophages (red) and DAPI to label nuclei (blue) (Figure 6F). The results revealed that compared to the Con group, the expression of CD206+ in the pancreas of T2DM mice was significantly increased (*p* < 0.01). Most importantly, after DP intervention, the expression of CD206+ in the pancreas increased by 2.09-fold compared to the Mol group and was significantly higher than in the Met group. These findings suggested that the infiltration of M2-type macrophages might be associated with the alleviation of local pancreatic inflammation, but its causality needs to be further verified.

### 3.7. Effects of DP on Gut Microbiota in T2DM Mice

**Figure 7:**
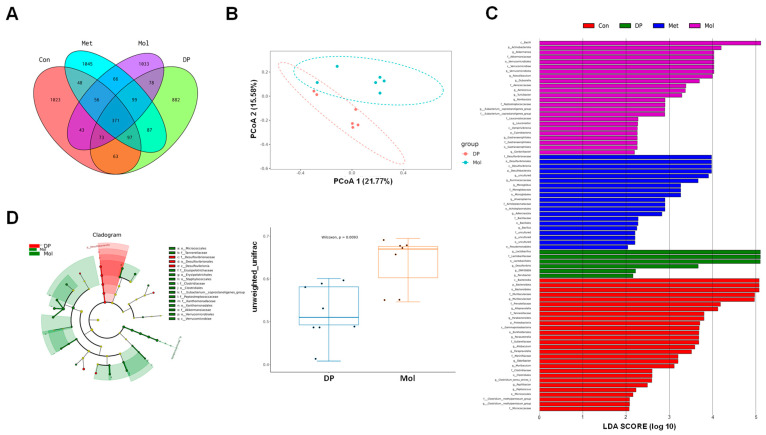
Effects of DP on the diversity of gut microbiota in mice with T2DM. (**A**) OTUs Venn graph of mice feces in each group. (**B**) Inter-group difference analysis of β-diversity based on Unweighted Unifrac distance and PCoA analysis diagram. (**C**) LDA scores of taxa enriched at different taxonomy levels. (**D**) Taxonomic cladogram generated by LEfSe analysis (n = 6).

We conducted 16S rDNA sequencing on fecal samples from each group of mice to determine the impact of DP on the composition of the gut microbiota. To identify taxa unique to different groups, a Venn diagram was used to cluster and partition operational taxonomic units (OTUs) at a 97% sequence similarity threshold. A total of 5064 OTUs were identified across all groups (Figure 7A). Among these, 1774, 1819, 1869, and 1750 OTUs were observed in the control group (Con), model group (Mol), metformin group (Met), and DP group, respectively. The DP group had 1750 OTUs, with 882 being unique.

For β-diversity analysis, intergroup differences analysis and principal coordinate analysis (PCoA) based on Unweighted Unifrac distances were performed (Figure 7B). The results showed that the microbial community structure was completely separated into two distinct clusters, with samples within each cluster being closely distributed. Intergroup differences were greater than intragroup differences (*p* < 0.01), indicating that HFD/STZ induction and DP intervention significantly altered the composition of the gut microbiota.

Additionally, linear discriminant analysis effect size (LEfSe) was used to highlight core bacterial phenotypes from phylum to genus that contributed to changes in microbiota composition (Figure 7C,D). At the phylum level, the Con group was enriched with *Bacteroidia* and *Parabacteroides*. At the family level, *Muribaculaceae*, *Prevotellaceae*, *Tannerellaceae*, *Sutterellaceae*, *Marinfilaceae*, *Clostridiaceae*, and *Micrococcaceae* were enriched. At the genus level, *Alloprevotella*, *Parabacteria*, and *Parasutterella* were enriched. Thus, these results suggested the potential role of DP in modulating microbiota abundance.

In the Mol group, *Actinobacteriota*, *Verrucomicrobiae*, and *Cyanobacteria* were enriched at the phylum level, while *Faecalibaculum*, *Dubosiella*, *Aerococcus*, *Turicibacter*, and *Romboutsia* were enriched at the genus level. These findings indicated that HFD/STZ induction reduced the enrichment of gut microbiota observed in the Con group. In the DP group, *Lactobacillaceae* was enriched at the family level, and *Lactobacillus*, *Desulfovibrio*, and *Parvibacter* were enriched at the genus level.

## 4. Discussion

Type 2 diabetes mellitus (T2DM) is a metabolic disorder characterized by chronic hyperglycemia due to insulin resistance in peripheral tissues and progressive dysfunction of β-cells in the pancreas. Insulin resistance (IR) is considered a precursor and primary pathogenic factor of T2DM [27]. The liver is a key target for insulin activity, and HepG2 cells are commonly utilized in vitro to study hepatic insulin resistance [28]. The transport of glucose across the plasma membrane is the rate-limiting step for glucose entry into cells. To enhance cellular glucose uptake, insulin signaling promotes the translocation of specific glucose transporters to the plasma membrane [29]. In this study, we induced insulin resistance in HepG2 cells and found that DP intervention significantly improved glucose uptake in the IR-HepG2 model. The IRS1/PI3K/AKT pathway is a major signaling pathway associated with insulin resistance, mediating insulin-stimulated glucose uptake and utilization. Insulin binds to its receptor, triggering the phosphorylation of insulin receptor substrates, which subsequently activates PI3K and its downstream effector AKT [30,31]. Serine phosphorylation of IRS1 can inhibit its tyrosine phosphorylation, blocking downstream pathways and impairing insulin signaling. GLUT4, a major insulin-responsive glucose transporter, can translocate from perinuclear compartments (including GLUT4 storage vesicles) to the plasma membrane [32,33]. To further explore the mechanism of DP, we assessed the levels of IRS1 and GLUT4 in the IR-HepG2 model before and after DP intervention. Our findings indicated that DP promoted glucose uptake by upregulating IRS1 and GLUT4, suggesting that DP may improve glucose metabolism by activating the IRS1/GLUT4 signaling pathway.

Prolonged consumption of a high-fat diet results in excessive fat storage and elevated free fatty acids in diabetic mice, leading to insulin resistance and hindered glucose metabolism [34]. A T2DM mouse model was created through HFD/STZ induction, with metformin serving as a reference treatment. DP intervention significantly reversed typical features of T2DM, including weight loss, elevated FBG, and abnormal oral glucose tolerance. Notably, the Met group exhibited sustained lower body weight, possibly attributed to the drug’s adverse effects that impeded weight restoration. Our previous studies have shown that DP can increase skeletal muscle strength and improve muscle mass and strength, which may significantly benefit T2DM patients experiencing weight loss [35]. Additionally, accelerated lipid breakdown during diabetes leads to abnormal levels of total cholesterol (TC) and triglycerides (TG) [36]. In this study, DP intake reduced serum levels of TC, TG, and LDL-C, while increasing levels of TP and ALB in T2DM mice. These findings indicate that DP can regulate lipid and protein metabolism while stabilizing glucose levels, thereby mitigating diabetic complications to some extent.

T2DM is frequently associated with hepatic steatosis, although the exact causal relationship between the two conditions remains uncertain. Hepatic lipid accumulation could serve as a potential indicator of T2DM [37]. Excessive intake of high-sugar and high-fat diets releases large amounts of fatty acids into the liver. When these exceed the liver’s clearance capacity, lipid deposition occurs, leading to hepatic steatosis [38]. Hepatic glycogen levels play a crucial role in monitoring glucose metabolism. The liver regulates glucose output and glycogen synthesis to maintain glucose homeostasis. Analysis of liver tissue samples from treated mice indicated a significant improvement in liver damage and an increase in hepatic glycogen content with DP treatment. This effect may be attributed to DP providing protein nutrition to T2DM mice, enabling damaged hepatocytes to engage their repair mechanisms and regain normal structural integrity. Additionally, the enzymes and coenzymes required for various reactions in hepatocytes were sufficiently supplied, allowing rapid restoration of liver function. Insulin regulates blood glucose levels by binding to its receptor and stimulating downstream proteins. T2DM is associated with disturbances in various glucose metabolism-related signaling pathways in the liver, notably the IRS1/PI3K/AKT pathway [29]. In the absence of abnormalities, insulin binding triggers tyrosine phosphorylation of IRS1, suppressing serine phosphorylation and initiating subsequent signaling cascades. Conversely, in insulin resistance, serine phosphorylation of IRS1 intensifies, impeding downstream signaling activation. Following intervention with DP, there was a partial recovery in the levels of IRS1 and PI3K proteins, indicating that DP might ameliorate hepatic insulin resistance by stimulating the IRS1/PI3K signaling pathway.

Islet cell damage is a hallmark of T2DM. Histological analysis of pancreatic tissues showed that DP effectively alleviated islet damage in T2DM mice. In addition to islet cell damage, T2DM disrupts insulin secretion regulation, leading to insufficient insulin production [39]. Our results showed that both metformin and DP interventions significantly promoted insulin secretion, consistent with findings by OGIWARA et al. [40]. Pancreatic inflammation is a critical component of T2DM pathophysiology [41]. In patients with T2DM, the number of macrophages in islets increased significantly, along with elevated levels of cytokines and chemokines. Additionally, increased expression of CD4+ cells and macrophages has been observed in the exocrine compartment [42]. These studies suggested that pancreatic inflammation accompanies T2DM. CD4+ and CD8+ T-cell subsets play a central role in the immune system, maintaining immune balance through mutual regulation. Nevertheless, persistent inflammation during the advancement of T2DM disrupts this equilibrium, resulting in metabolic irregularities and the accumulation of metabolic byproducts, thereby intensifying immune dysfunction and disease progression [43]. CD3+ T-cell activation markers (CD4+ and CD8+) in pancreatic tissues of HFD/STZ-induced T2DM mice analyzed by flow cytometry revealed that DP reduced the proportion of CD4+ cells compared to T2DM mice, although not significantly, suggesting potential immune-modulating effects. During inflammation, the number of immune-related cells increases. Studies have shown that CD45+ leukocyte infiltration in islets was higher in T2DM patients compared to healthy individuals [44]. Reducing leukocyte infiltration may help alleviate inflammation. Flow cytometry and immunofluorescence analysis showed a significant reduction in CD45+ cell infiltration after DP intervention. Previous studies have highlighted the role of protein nutrition in reducing inflammation, consistent with our findings [45]. These results indicated that DP can significantly suppress pancreatic inflammation and reduced CD45+ infiltration. Macrophages, as innate immune cells, can polarize into M1 or M2 phenotypes under physiological and pathological conditions. T2DM-induced hyperglycemia promoted M1 polarization and inhibited M2 polarization, thus aggravating inflammation and disrupting immune balance [46]. Therefore, redirecting and reshaping macrophage polarization is considered a therapeutic strategy for T2DM. Our findings indicated that DP intervention significantly elevated CD206+ expression in pancreatic tissue. Consequently, we speculated that the infiltration of M2-type macrophages might be associated with the alleviation of local pancreatic inflammation, but its causality needs to be further verified.

As understanding of the gut microbiota deepens, increasing evidence suggests its role in metabolic disorders such as obesity and T2DM. Dysbiosis of the gut microbiota increases intestinal permeability, allowing bacterial endotoxins to enter the bloodstream, triggering inflammation, insulin resistance, and hyperglycemia [47]. To elucidate the effects of DP supplementation on gut microbiota, we examined changes in microbial composition and bacterial relative abundance in treated mice. In this study, DP supplementation altered the gut microbiota composition in T2DM mice. Different bacteria exert varying effects on host homeostasis. Notably, DP increased the abundance of *Lactobacillus*, *Parvibacter*, and *Lactobacillaceae*. The enrichment of *Lactobacillus* may be associated with plant-based protein intake, which is closely linked to the development of chronic metabolic diseases, particularly diabetes [48]. Studies have shown that Lactobacillus treatment can alleviate FBG levels, postprandial glucose, glucose tolerance, and liver damage in HFD/STZ-induced diabetic mice. The anti-diabetic potential of Lactobacillus may involve regulating glucose and lipid metabolism, energy metabolism, and reducing systemic inflammation [49]. *Parvibacter*, a naturally occurring commensal bacterium, constitutes 3–5% of the healthy human gut microbiome. Numerous microbiome analyses have shown reduced *Parvibacter* abundance in various disease states, including irritable bowel disease, Crohn’s disease, asthma, depression, and metabolic disorders [50]. Specifically, reduced *Parvibacter* levels are associated with elevated blood glucose and T2DM. To sum up, our findings indicated that DP could prevent T2DM via repairing gut microbiota.

## 5. Conclusions

In this study, we investigated the effects of DP on the HepG2 insulin resistance cell model at the in vitro level. The findings demonstrated that DP increased glucose uptake by potentially modulating the IRS1/GLUT4 signaling pathway, thus alleviating insulin resistance in HepG2 cells. In the T2DM mice model, nutritional intervention with DP significantly enhanced glucose, lipid, and protein metabolism, reduced inflammation in HFD/STZ-induced T2DM mice, and improved liver and pancreatic function. These effects were potentially mediated through the activation of the Insulin/IRS1/PI3K signaling pathway and modulation of gut microbiota composition and abundance. In conclusion, these results suggest that soy–whey dual-protein supplementation may ameliorate hepatic insulin resistance and T2DM, offering a novel and promising nutritional intervention for T2DM patients to mitigate inflammatory responses and protect organ function. Further studies will be conducted in the future, including dose-response studies to optimize therapeutic regimes, metabolic profiling to clarify microbiota-mediated mechanisms, and explorations of alternative pathways. In addition, targeted clinical trials can also provide more powerful evidence for the application of dual-protein in the prevention and treatment of diabetes.

## Data Availability

Data is provided within the manuscript information files.

## Supplementary Materials

The following supporting information can be downloaded at: https://www.mdpi.com/article/10.3390/foods14122115/s1, Table S1: Amino Acid Profile of Dual-Protein.

## Abbreviation

DP, dual-protein; T2DM, type 2 diabetes mellitus; HFD, high-fat diet; STZ, streptozotocin; IR, Insulin resistance; IRS1, Insulin receptor substrate 1; GLUT4, Glucose transporter 4; CLSM, Confocal laser scanning microscopy; FBG, Fasting blood glucose; INS, Insulin; LDL, Low density lipoprotein; OGTT, Oral glucose tolerance test; PBS, Phosphate buffered solution; PI3K, Phosphatidylin-ositol-3-kinase; TC, Total cholesterol; TG, Triglyceride; TP, Total protein; ALB, Albumin.
