# Supplementary material for: Anti-Diabetic Effect of Soy–Whey Dual-Protein on Mice with Type 2 Diabetes Mellitus Through INS/IRS1/PI3K Signaling Pathway
Source: Foods. 2025 Jun 16;14(12):2115. doi: 10.3390/foods14122115 (PMC12192230; doi:10.3390/foods14122115)
Supplement: Supplementary file 1 [file foods-14-02115-s001.zip › foods-3672706-supplementary.pdf]

Supplementary table

Amino acids	Content [g/100g]
Aspartic acid	101.654
Threonine	49.752
Serine	49.494
Glutamic acid	150.662
Glycine	27.193
Alanine	47.926
Cystine	2.445
Valine	50.243
Methionine	29.34
Isoleucine	58.327
Leucine	92.823
Tyrosine	43.718
Phenylalanine	48.541
Histidine	28.263
Lysine	85.995
Arginine	53.995
Total	920.37

Table S1: amino acid profile of dual-protein.
